# Loop-Mediated Isothermal Amplification Coupled With Nanoparticle-Based Biosensor: A Rapid and Sensitive Method to Detect *Mycoplasma pneumoniae*

**DOI:** 10.3389/fcimb.2022.882855

**Published:** 2022-07-06

**Authors:** Fei Xiao, Juan Zhou, Chunrong Sun, Xiaolan Huang, Baoying Zheng, Jin Fu, Nan Jia, Zheng Xu, Xiaodai Cui, Yi Wang

**Affiliations:** ^1^ Experimental research center, Capital Institute of pediatrics, Beijing, China; ^2^ Department of Respiratory Disease, Capital Institute of pediatrics, Beijing, China

**Keywords:** *Mycoplasma pneumonia*e, loop-mediated isothermal amplification, lateral flow biosensor, community-acquired respiratory distress syndrome, MP pneumonia, MP pneumonia

## Abstract

*Mycoplasma pneumoniae* (*MP*), the causative agent of *MP* pneumonia (MPP), has posed a substantial burden to public health owing to a lack of rapid and effective diagnostic methods. Here, we designed a loop-mediated isothermal amplification (LAMP)-based assay, termed LAMP, combined with a nanoparticle-based lateral flow biosensor (LAMP-LFB) for rapid and sensitive diagnosis of *MP.*-LAMP-LFB included a set of six primers targeting the community-acquired respiratory distress syndrome (CARDS) toxin gene and was performed optimally at 63°C for only 30 min. The resulting LAMP products could be visually indicated by LFB within 2 min, thus the whole process could be accomplished within an hour. *MP*-LAMP-LFB’s sensitivity was 50 fg per reaction, which was in complete accordance with these results obtained from real-time turbidity and visual detection reagent (VDR). *MP*-LAMP-LFB had no cross-reactivity with other pathogens that had similar clinical presentations. Our assay was further validated using 100 nasopharyngeal swab samples collected from children suspected of MPP, and the result was compared with the real-time PCR method. With a positive rate of 50%, the data indicated that *MP*-LAMP-LFB is a sensitive test for *MP* detection in clinical settings. Collectively, the *MP*-LAMP-LFB assay targeting the CARDS toxin gene was a rapid, highly sensitive, and specific test that could be widely applied in point-of-care settings and basic medical facilities in rural areas.

## Introduction

*Mycoplasma pneumoniae* (*MP*) is one of the most common pathogens of community-acquired pneumonia (CAP), with school-age children and adolescents being most easily affected (Winchell, 2013). *M. pneumoniae* may epidemically cause up to 40% of community-acquired bacterial pneumonias (CABP) among general populations, and 70% in closed populations (Loens et al., 2010; Jacobs et al., 2015; Li et al., 2022). However, the true impact of this organism on public health is underestimated for its benign nature and lack of specific clinical presentations and excellent diagnostic method (Waites et al., 2017). Thus, the development of a simple, rapid, and accurate laboratory detection method of *M. pneumoniae* is especially important for accurate clinical diagnosis, effective treatment, and early surveillance.

Traditional detection methods of *M. pneumoniae* infection are mainly based on culture, serological testing, and PCR-based methods. Culture is recognized as the “gold standard” of *M. pneumoniae* infection when positive (100% specificity), however, the disadvantages of expensive apparatus; time-consuming, laborious, requirement of additional procedures for species identification; and low sensitivity limited its wide clinical application, especially in acute care settings (Waites et al., 2017). Serological detection, targeting on the antibodies IgM and/or IgG against *M. pneumoniae* in serum specimens, is the main method for diagnosis and epidemiological investigation of *M. pneumoniae*. However, the timing of specimen collection and the performance characteristics of the selected commercial kit substantially affects the sensitivity and specificity of this method (Waites et al., 2017). Moreover, weak immune response of infants and the elderly as well as potential cross-reactivity with other pathogens and a single measurement of the antibodies are also influencing factors of false-negative as well as false-positive results (Chang et al., 2014; Medjo et al., 2014; Bajantri et al., 2018). PCR-based diagnostic techniques have been considered as the “new gold standard” because of the superior analytical and clinical sensitivity and shorter turnaround time when compared with methods mentioned above (Waites et al., 2012; Loens and Ieven, 2016). However, the exact sensitivity and specificity of these PCR-based methods varies with the corresponding amplification platforms and detection formats (Waites et al., 2017). Moreover, the requirements of complex equipment and trained personnel of PCR-based methods make them more difficult to be widely used for a point-of-care test (Li et al., 2020).

In addition to PCR-based molecular method, the loop-mediated isothermal amplification (LAMP) technology, another relatively new nucleic acid amplification test format, is widely used for direct detection of clinically important etiologic agents including *M. pneumoniae* (Parida et al., 2008; Kakuya et al., 2014; Baek et al., 2020). LAMP method amplifies the target DNA under isothermal conditions with six oligonucleotide primers located at different regions of the target sequence and a strand displacement DNA polymerase, and the result is detected by measuring the turbidity change in the reaction mixture, which is caused by the combination of the by-product of pyrophosphate ions with Mg^2+^ (Notomi et al., 2000). With favorable characteristics in terms of diagnostic sensitivity, specificity, rapidity, and simplicity, LAMP technology is now regarded as the first-line for diagnosing acute *M. pneumoniae* infections in Japan (Kakuya et al., 2014). However, traditionally, detection of the turbidity change in the reaction mixture needs the help of spectrophotometric apparatus, which will limit their wide use in condition-restricted settings (Wang et al., 2019). Although visual detection reagent (VDR) is an alternative, the results become less accurate under low template concentration.

Nanoparticles-based lateral flow biosensor (LFB) is a kind of paper-based device with advantages of good robustness, rapidity, specificity, sensitivity, low limits of detection, as well as cost-effectivity (Li et al., 2020). Moreover, detection of different types of compounds with LFB permits the readout visually and independent of sophisticated apparatus (Wang et al., 2019). Therefore, in this study, a new method coupling LAMP technology with LFB is developed for simply, quickly, intuitively, and objectively diagnosing *M. pneumoniae* infection without limitation of equipment or experience. The newly proposed *M. pneumoniae*-LAMP-LFB assay used the community-acquired respiratory distress syndrome (CARDS) toxin gene as the target, and was validated by pure cultures and clinical specimens.

## Materials and Methods

### Reagents and Instruments

Both common and labeled primers used in this study were synthesized by AOKE Biotech Co., Ltd (Beijing, China). VDR, DNA Isothermal Amplification Kit, and LFB were all provided by HUIDEXIN Biotech Co., Ltd (Tianjin, China). Genomic DNA kit for nucleic acid extraction and purification was purchased from Beijing TransGen Biotech Co., Ltd (Beijing, China). Real-time PCR kit was purchased from Sansure BioTech Co., Ltd (Changsha, China) (http://www.sansure.com.cn/). Real-time turbidimeter LA-320C was purchased from Eiken Chemical Co., Ltd, Japan.

### Primer Design

**Figure 1:**
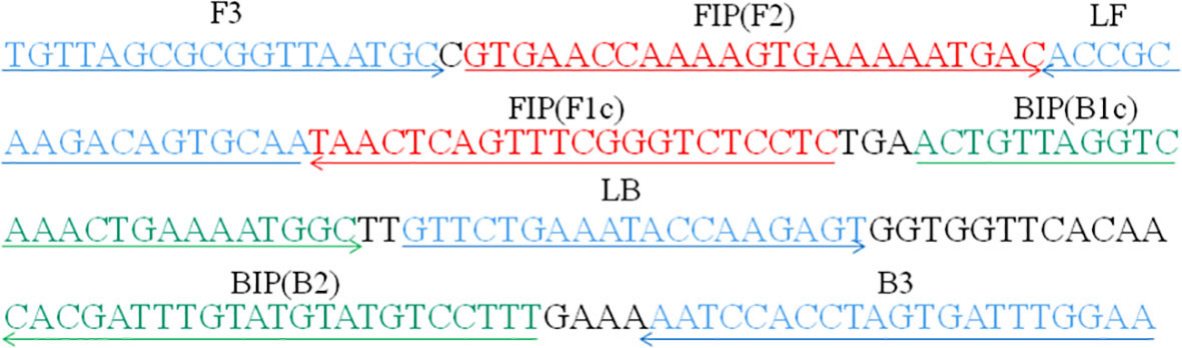
Primers specific to the CARDS toxin gene of *M. pneumoniae* used for LAMP-LFB assay in this study. Locations, sequences, and directions of the LAMP-LFB primers are displayed. Right and left arrows indicate sense and complementary sequences, respectively.

A set of six primers spanning eight different regions of the CARDS toxin gene in M. pneumoniae, including two outer primers (F3 and B3), two inner primers (FIP/FIP* and BIP), and two loop primers (LF* and LB), were designed using PrimerExplorer 5 (https://primerexplorer.jp). Following subjected to blast against the NCBI database, the primers that nonspecifically matched with other microorganisms were excluded and the optimal ones were achieved. The sequences, locations, and modifications of the primers used in this report are shown in **Figure 1** and **Table 1**.

**Table 1:** Sequences and modifications of the LAMP-LFB primers.

Primers ^a^	Sequence (5’-3’) and and modifications	Length ^b^
F3	TGTTAGCGCGGTTAATGC	18 nt
B3	TTCCAAATCACTAGGTGGATT	21 nt
FIP	GAGGAGACCCGAAACTGAGTTA-GTGAACCAAAAGTGAAAAATGAC	46 mer
FIP*	Biotin-GAGGAGACCCGAAACTGAGTTA-GTGAACCAAAAGTGAAAAATGAC	46 mer
BIP	ACTGTTAGGTCAAACTGAAAATGGC-AAAGGACATACATACAAATCGTG	49 mer
LF	TTGCACTGTCTTGCGGT	17 nt
LF*	FITC-TTGCACTGTCTTGCGGT	21 nt
LB	GTTCTGAAATACCAAGAGT	19 nt

a F3, forward outer primer; FIP, forward inner primer; LF, Forward loop primer; LB, Backward loop primer; BIP, backward inner primer; B3, backward outer primer; FIP*, 5’-labeled with biotin when used in LAMP-LFB assay; LF*, 5’-labeled with carboxyfluorescein when used in LAMP-LFB assay. FIP and LF primers were labeled to obtain LAMP amplifiers on the turbidimeter and detect signals in the LFB test at the same time.

b nt, nucleotide; mer, monomeric unit.

### The Standard LAMP-LFB Assay

The DNA sample of *M. pneumoniae* was extracted and used as positive control to establish the standard LAMP-LFB assay. The LAMP assay was performed in a 25 μL reaction mixture containing 12.5 μL 2 × reaction buffer, 0.1 μM F3 and B3 (each), 0.4 μM FIP* and BIP (each), 0.2 μM LF* and LB (each), 1.0 μL *Bst* DNA polymerase (8 U), 1.2 μL VDR, 1.0 μL template (5.0 μL in sample detection), and 7.3 μL distilled water (DW). The mixtures were incubated at 64°C for 1 h, and then at 80°C for 5 min to terminate the reaction. Mixture with 1.0 μL genomic DNA of *Haemophilus influenzae* was used as negative control, and the one with 1 µL DW as blank control.

Monitoring techniques, including colorimetric indicator (visual detection reagent, VDR), real-time turbidity (LA-320C), and LFB detection, are employed for confirming and validating the *Mycoplasma pneumoniae*-LAMP assay. Particularly, VDR and real-time turbidity were used for validating the results obtained from LFB detection. For VDR, a color change (light green) of positive *Mycoplasma pneumoniae*-LAMP reactions was directly observed with unaided eyes after isothermal amplification. For LFB indication of LAMP results, the details have been reported in previous reports (Wang et al., 2017; Li et al., 2019). In brief, a 2 µL aliquot of *Mycoplasma pneumoniae*-LAMP products, which were biotin- and FITC-labeled amplicons, was loaded into the sample zone. Then, a volume of 80 µL of running buffer was also loaded into the sample zone. Thus, the *Mycoplasma pneumoniae*-LAMP amplicons were specifically captured by the immobilized anti-FITC at the first test line, and detector reagents rapidly accumulated in the first test line by streptavidin/biotin interaction, resulting in a visual red colored band. The proper function of LFB is validated by the second line (control line) formation which captured excess detector reagent through biotinylated bovine serum albumin. As a result, two visible red lines (test line and control line) were observed in positive amplifications, and only a visible red line (control line) was seen in negative amplifications (e.g., negative control, blank control).

### Optimize Temperature of the *M. pneumoniae*-LAMP Assay

Temperatures ranging from 60°C to 67°C (with an interval of 1°C) were examined for determining the optimal temperature using the standard LAMP reaction system. The amplified products were detected with real-time turbidity using the Real-time Turbidimeter LA-320, with a threshold value of > 0.1 within 1 h as positive reaction and a mixture with DW as blank control.

### Specificity of the *M. pneumoniae* -LAMP-LFB Assay

A total of 66 strains, including 11 *M. pneumoniae* strains, 11 other *Mycoplasma* strains, and 44 strains of other species (**Table 2**) were used to evaluate the analytical specificity of the *M. pneumoniae*-LAMP-LFB assay. The genomic DNA of all the strains were extracted according to the manufacturer’s instruction. The genomic copies of each tested strain should be the same and each test was repeated twice. The extracted genomic templates were determined with ultraviolet spectrophotometer (NanoDrop 2000, Thermo Fisher, Waltham, MA, USA) at A260/280, and extracted DNA was tested for concentration using NanoDrop2000 to achieve the same concentration.

**Table 2:** Strains for specificity confirmation of LAMP-LFB assay.

Species	Strain no. (source of the strains)^a^	No. of strains	*M. pneumoniae*-LAMP-LFB^b^
*Mycoplasma pneumoniae*	Isolated strains (CDC)	11	P
*Acinetobacter baumannii*	Isolated strains (CIP)	1	N
*Bacillus cereus*	Isolated strains (CDC)	1	N
*Citrobacter* spp.	Isolated strains (CDC)	2	N
*Corynebacterium sriatum*	Isolated strains (CDC)	1	N
*Enteroaggregative Escherichia coli*	Isolated strains (CDC)	1	N
Enteroinvasive *Escherichia coli*	Isolated strains (CDC)	1	N
Enteropathogenic *Escherichia coli*	Isolated strains (CDC)	1	N
Enterotoxigenic *Escherichia coli*	Isolated strains (CDC)	1	N
Shiga toxin-producing *Escherichia coli*	Isolated strains (CDC)	1	N
Enterococcus faecalis	Isolated strains(CDC)	2	N
*Haemophilus influenzae*	Isolated strains (CIP)	1	N
*Klebsiella pneumoniae*	Isolated strains (CDC)	3	N
*Listeria innocua*	Isolated strains (CDC)	1	N
*Listeria ivanovii*	Isolated strains (CDC)	1	N
*Listeria monocytogenes*	Isolated strains (CDC)	1	N
*Monilia albican*	Isolated strains (CDC)	2	N
*Moraxella catarrhalis*	Isolated strains (CDC)	1	N
*Mycobacterium tuberculosis*	Isolated strains (CDC)	2	N
*Neisseria meningitidis*	Isolated strains (CDC)	1	N
*Nocardia asteroides*	Isolated strains (CDC)	1	N
*Pseudomonas aeruginosa*	Isolated strains (CDC)	4	N
*Salmonella* spp.	Isolated strains (CDC)	2	N
*Shigella baumannii*	Isolated strains (CDC)	1	N
*Shigella sonnei*	Isolated strains (CDC)	1	N
*Staphylococcus amber*	Isolated strains (CDC)	1	N
*Staphylococcus epidermidis*	Isolated strains (CDC)	1	N
*Staphylococcus haemolyticus*	Isolated strains (CDC)	1	N
*Stenotrophomonas maltophilia*	Isolated strains (CDC)	1	N
*Steptococcus salivarius*	Isolated strains (CDC)	1	N
*Streptococcus aureus*	Isolated strains (CDC)	1	N
*Streptococcus pneumoniae*	Isolated strains (CDC)	1	N
*Streptococcus pyogenes*	Isolated strains (CDC)	1	N
*Streptococcus suis*	Isolated strains (CDC)	2	N
*Mycoplasma genitalium*	Isolated strains (#)	2	N
*Mycoplasma penetrans*	Isolated strains (#)	2	N
*Mycoplasma hominis*	Isolated strains (#)	2	N
*Mycoplasmaprimatum*	Isolated strains (#)	2	N
*Mycoplasma urealyticum*	Isolated strains (#)	3	N

^a^CIP, Capital Institute of Pediatrics; CDC, Chinese Center for Disease Control and prevention, # Beijing Children’s Hospital,

^b^P, positive; N, negative.

### Sensitivity of the *M. pneumoniae*-LAMP-LFB Assay

The genomic DNA of *M. pneumoniae* was serially diluted (5 ng, 500 pg, 50 pg, 5 pg, 500 fg, 50 fg, and 5 fg per microliter) to determine the sensitivity of the *M. pneumoniae*-LAMP-LFB assay. To measure the limit of detection (LoD), 1 μL of each serial dilution was added to the LAMP reaction mixtures and tested in duplicate. The lowest concentration of genomic DNA of *M. pneumoniae* was regarded as the LoD of the LAMP-LFB assay when both the duplicate samples were positive. LAMP reactions were recorded by real-time turbidity, LFB platform, and the VDR simultaneously.

### Optimize Amplification Time of *M. pneumoniae*-LAMP–LFB Assay

The serially diluted genomic DNA of *M. pneumoniae* (5 ng, 500 pg, 50 pg, 5 pg, 500 fg, 50 fg, and 5 fg per microliter) were used as a template and the procedure of amplification referred to the standard LAMP-LEB assay with 63°C as the reaction temperature. Reaction products were continually reported from the first 10 min to 40 min with an interval 10 min using VDR and LFB. The amount of time spent for the LoD template being positive was taken as the optimal amplification time. Each reaction was tested at least two replicates.

### Application of *M. pneumoniae*-LAMP-LFB Assay in Clinical Specimens

A total of 100 nasopharyngeal swab (NPS) samples, which were obtained from children suspected of *M. pneumonia* infection in the clinics of the Children’s Hospital affiliated to Capital Institute of Pediatrics from February 1 to December 30, 2021, were enrolled in the study for further evaluation of the LAMP-LFB assay. The ethical practice was approved by the Ethical Committee of Capital Institute of Pediatrics, and all the samples were obtained with informed consents signed by the participants’ guardians. Nucleic acids extracted from these samples were used for clinical and laboratory diagnosis for the first time. All samples were detected by using both LAMP-LFB assay and real-time PCR assay, and the results were compared with each other. Particularly, the commercial real-time PCR kit, which was obtained from Sansure Biotech Co., Ltd (Changsha, China), was employed for *M. pneumonia* detection.

### Statistical Analysis

A comparison between the two methods, real-time PCR and LAMP-LFB assay, were analyzed by χ^2^ test with SPSS software (version 11.5) and *P <*0.05 was considered statistically significant.

## Results

### Confirmation of Effectiveness of LAMP-LFB Assay for the Detection of *M. pneumoniae*

**Figure 2:**
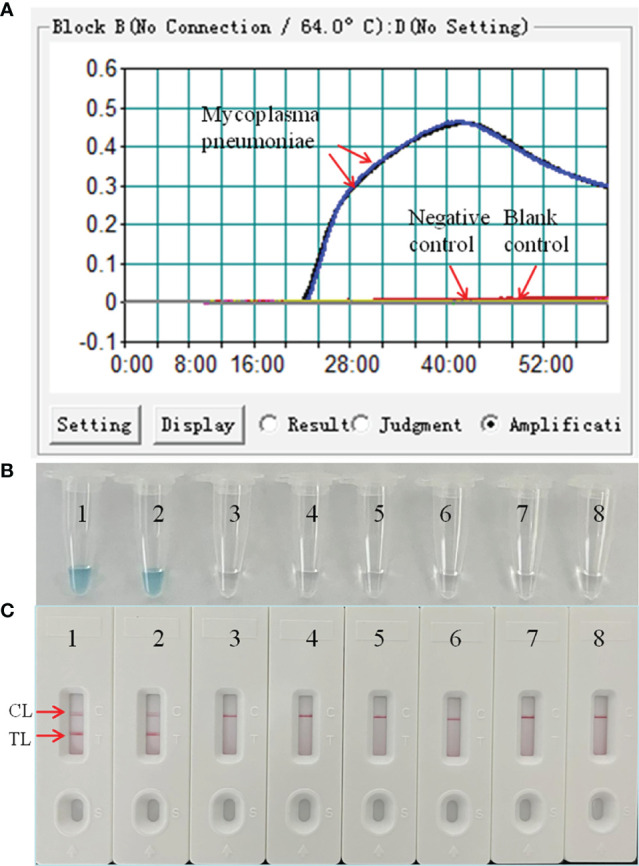
Confirmation and detection of *M. pneumoniae*-LAMP-LFB amplification products by real-time turbidity detection. The DNA templates extracted from *M. pneumoniae* strains were effectively amplified with LAMP reaction at 64°C, and there’s no obvious amplification in the negative and blank controls. The products were indicated real-time turbidity **(A)**, the visual detection reagent **(B)**, and a nanoparticle-based lateral flow biosensor (LFB) test **(C)**. Tubes/strips 1-2 represent DNA templates extracted from *M. pneumoniae* strains. Tube/strip 3-6 represent DNA templates extracted from *Haemophilus influenzae*, Tube/strip 7-8 represent the blank control. CL, control line; TL, test line.

The LAMP reaction was performed at 64°C for 60 min to validate the feasibility of the designed primers. Using real-time turbidimeter, a significant increase of turbidity was observed in the mixture with *M. pneumoniae* genomic DNA as template, while an almost blunt curve was seen in the negative and blank control (**Figure 2A**). Using VDR, the color shift of positive results in LAMP tubes from colorless to light green was observed with naked eyes after incubation (**Figure 2B**), and negative results in LAMP tubes remained colorless. Using LFB, two red bands (CL and TL) were visible in positive results, but only a line (CL) was seen in negative and blank controls (**Figure 2C**). These data above suggested that the selected primers were effective-sufficient for detection of *M. pneumoniae* using LAMP-based assay.

### Optimal Temperature of *M. pneumoniae*-LAMP-LFB Assay

**Figure 3:**
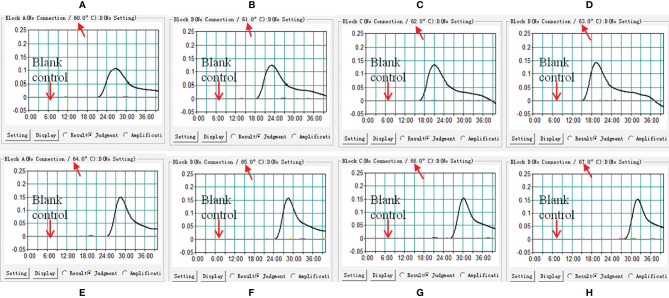
Confirmation of optimal reaction temperature of *M. pneumoniae*-LAMP assay. The amplified products of *M. pneumoniae*-LAMP assay were read out by real-time turbidimeter, and the corresponding curves of each temperature were displayed in the picture. Turbidity > 0.1 was considered as positive results. Eight kinetic graphs **(A–H)** were acquired at different temperatures ranging from 60 to 67°C with a 1°C interval.

In this report, we performed LAMP-LFB assay at eight different temperatures ranging from 60 to 67°C at 1°C intervals for 40 min to optimize the temperature. As shown in **Figure 3**, 63°C was the optimal temperature for *M. pneumoniae*-LAMP-LFB amplification because 63°C was the temperature at which the threshold value of 0.1 of absorbance was achieved fastest. Therefore, 63°C was used for the subsequent *M. pneumoniae*-LAMP-LFB reaction conducted in this report.

### Specificity of the LAMP-LFB Assay for the Detection of *M. pneumoniae*

DNA templates of *M. pneumoniae* and non-*M. pneumoniae* strains were used to estimate the specificity of the *M. pneumoniae-*LAMP-LFB assay under the optimal conditions confirmed above. Using LFB, two red lines were seen at the location of both TL and CL for the *M. pneumoniae* strain, and only one line was seen at the location of CL for all the non-*M. pneumoniae* strains and blank control (**Figure 6**), suggesting that the specificity of the *M. pneumoniae*-LAMP-LFB assay was 100%.

### Sensitivity of the LAMP-LFB Assay for the Detection of *M. pneumoniae*

**Figure 4:**
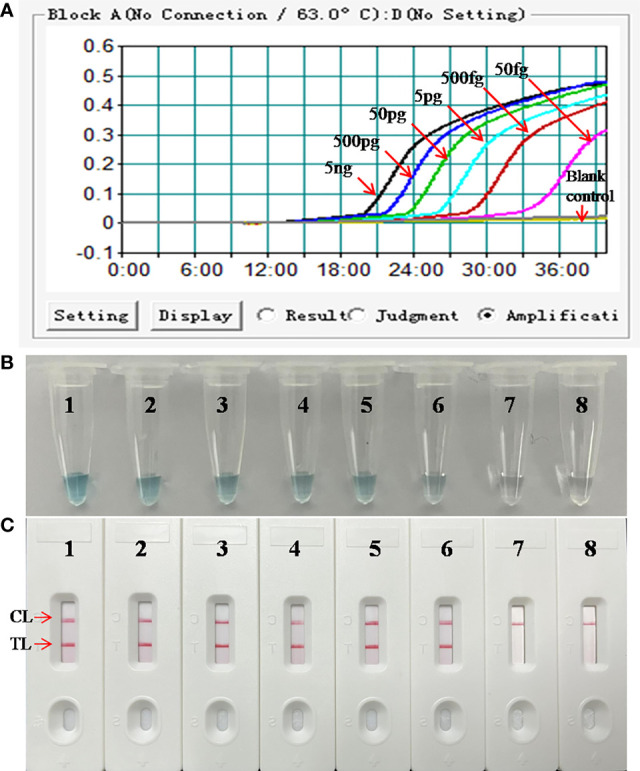
Analytical sensitivity of *M. pneumoniae*-LAMP assay. Three detection formats: **(A)** real-time turbidity; **(B)** colorimetric indicator; **(C)** LFB were used for amplified products analysis. Serial dilutions (5 ng, 500 pg, 50 pg, 5 pg, 500 fg, 50 fg, and 5 fg) of genomic DNA of *M. pneumoniae* were used for LoD determination of LAMP assay. Strips/tubes 1-7 represented genomic DNA at the level of 5 ng, 500 pg, 50 pg, 5 pg, 500 fg, 50 fg, and 5 fg, respectively; strip/tube 8, blank control. CL, control line; TL, test line. The DNA templates of *M. pneumoniae* from 5 ng to 50 fg produced positive results. CL, control line; TL, test line.

The DNA templates of *M. pneumoniae* were serially diluted for the examination of the LoD of the assay. As shown in **Figure 4**, the results detected by the LFB showed that the LoD of the *M. pneumoniae-*LAMP-LFB was as low as 50 fg (~12 copies) per reaction, in accordance with those indicated by turbidity and VDR.

### Optimal Time of the *M. pneumoniae*-LAMP-LFB Assay

**Figure 5:**
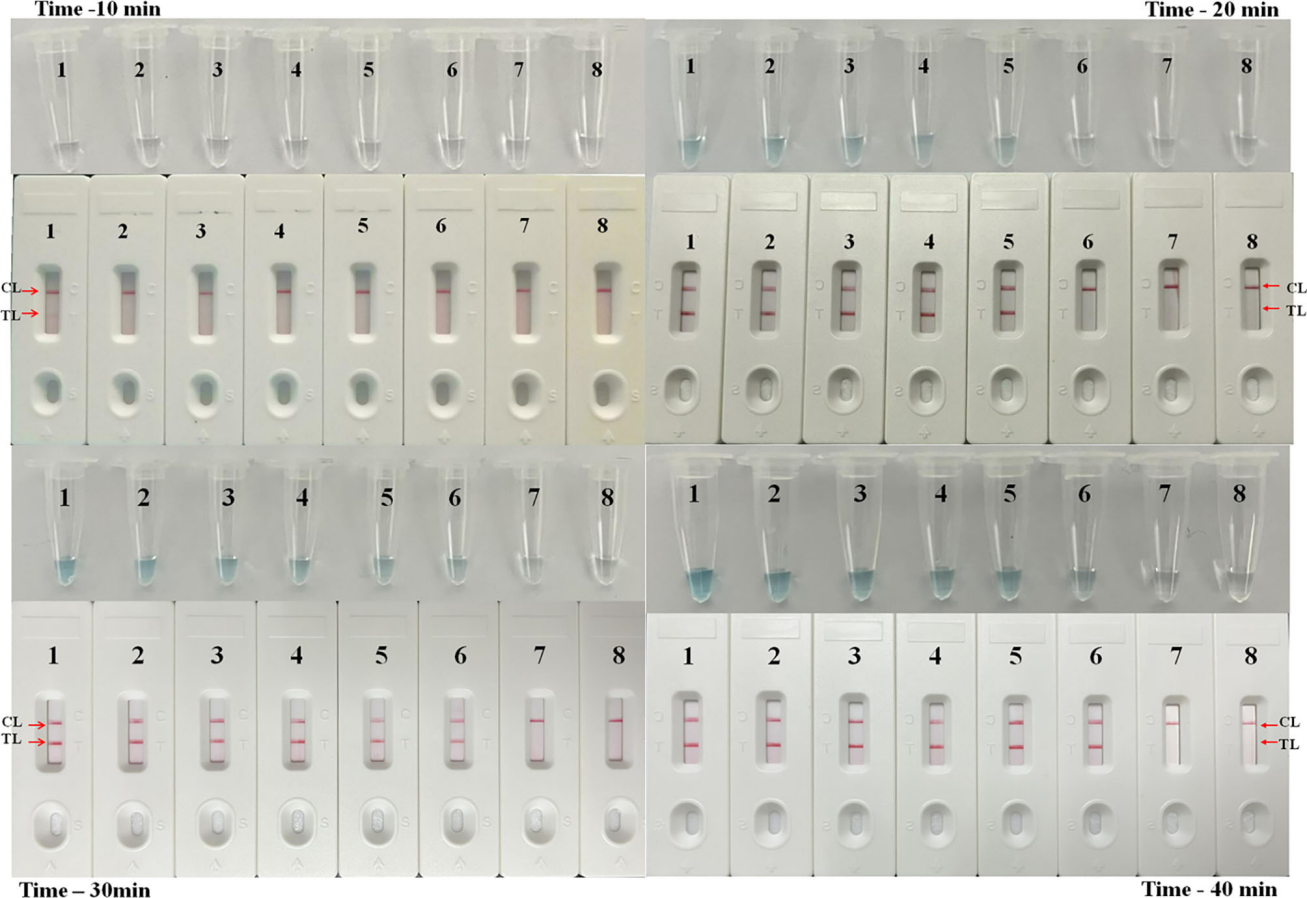
Time optimization for *M. pneumoniae*-LAMP assay. The standard LAMP reaction with four distinct times including 10 min, 20 min, 30 min, and 40 min were tested and analyzed by LFB and VDR at the optimal temperature of 63°C. Strips/tubes 1-7 represented genomic DNA at the level of 5 ng, 500 pg, 50 pg, 5 pg, 500 fg, 50 fg, and 5 fg, respectively; strip/tube 8, blank control. CL, control line; TL, test line.

Reaction products of the LAMP assay were tested by using the LFB platform every 10 min from the 10th to the 40th min at 63°C to confirm the optimal amplification time of *M. pneumoniae*-LAMP-LFB assay. As shown in **Figure 5**, after reaction for 30 min, the products of the tube with template at the LoD level was detectable, indicating that 30 min was enough for the *M. pneumoniae*-LAMP assay. Hence, the whole procedure of *M. pneumoniae*-LAMP-LFB analysis only requires approximately 60 min for target pathogen detection, which includes rapid DNA extraction (25 min), isothermal reaction (30 min), and result indication (2 min).

### Application of *M. pneumoniae*-LAMP-LFB Assay in Clinical Specimens

**Figure 7:**
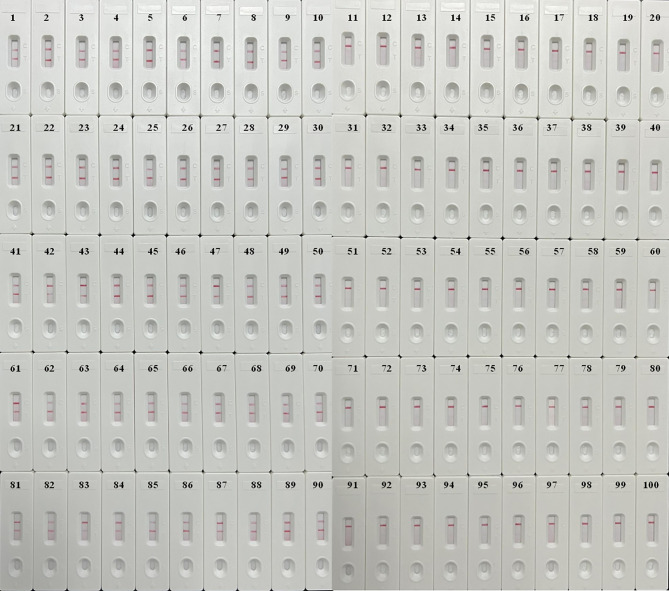
Validation of *M. pneumoniae*-LAMP-LFB assay in clinical specimens. Results of *M. pneumoniae*-LAMP-LFB assay for 100 nasopharyngeal swab samples from children suspected of *M. pneumonia* infection showed that 50 samples tested positive.

In order to confirm the availability of clinical application, the optimized *M. pneumoniae-*LAMP-LFB assay was used to detect 100 NPS samples, which were concurrently detected by real-time PCR method. Results showed that 50 (50%) samples tested positive by the LAMP-LFB assay, and 47 (47%) samples were positive by real-time PCR (**Table 3**) (**Figure 7**). Both methods had good concordance of 94% with a kappa value of 0.94 and a *p*-value of <0.001.

**Table 3:** Comparison of LAMP-LFB assay and real-time PCR for *M. pneumoniae* detection in clinical specimens.

Detection assay	Nasopharyngeal swab samples	
	Positive	Negative
LAMP-LFB	50 (50%)	50 (50%)
Real-time PCR	47 (47%)	53 (53%)

## Discussion

LAMP technology, first proposed by Notomi *et al.* in 2000 (Notomi et al., 2000), is a relatively new nuclei acid amplification-based assay for direct detection of a variety of pathogenic microorganisms including bacteria, virus, and fungi (Endo et al., 2004; Ziros et al., 2015; Wang et al., 2017; Wang et al., 2019), and can couple with different detection formats for product confirmation such as agar gel electrophoresis, real-time turbidimeter, colorimetric indicator, and LFB. Among all the detection formats, LFB is a potential contender for its traits of portability, easy to operation, and fast read-out: agarose gel electrophoresis and real-time turbidimeter depend on complicated and expensive apparatus and experienced personnel; colorimetric indicator is somewhat subjective, especially at a low concentration, although it is simple and cheap (Wang et al., 2019). Coupling with LFB, the LAMP-LFB assay is more applicable for bedside diagnosis or in a shabby environment because of its independence of expensive facilities or experienced personnel. With only an isothermal heater or water bath, the LAMP-LFB assay could provide favorable results in terms of speediness, efficiency, sensitivity, specificity, and simplicity, as demonstrated in the detection of SARS-CoV-2, *Brucella* spp., *Enterococcus faecalis*, *Staphylococcus aureus*, and so on (Wang et al., 2017; Li et al., 2019; Zhu et al., 2020).

The LAMP-LFB assay also has been previously developed for the diagnosis of *M. pneumoniae* infection, with P1 gene as the target (Wang et al., 2019). Aside from the P1 gene, other genes have also been studied for the detection of *M. pneumoniae* by using other nuclei acid amplification formats, such as 16S rRNA, the 16S-23S rRNA spacer, the CARDS toxin gene, the ATPase operon, *dnaK*, *pdhA*, *tuf*, *are*, *pdhA*, *ptsL*, and the noncoding repetitive element in repMp1 (Waites et al., 2017). It is noteworthy that the CARDS toxin gene was reported to be more sensitive than the P1 gene with 10-fold more patients being diagnosed by using PCR (Peters et al., 2011). As a pathogen that could induce infections both endemically and epidemically but lacking specific clinical presentations or laboratory assessments, early and accurate detection of *M. pneumoniae* is of ultimate significance for the prevention and treatment of *M. pneumoniae* infection. In this study, a LAMP-LFB assay targeting the CARDS toxin gene was established and evaluated for *M. pneumoniae* detection in pure cultures and clinical specimens. The results showed that this assay is of high sensitivity and specificity and could be accomplished within an hour, indicating its very wide popularization and application prospects.

**Figure 6:**
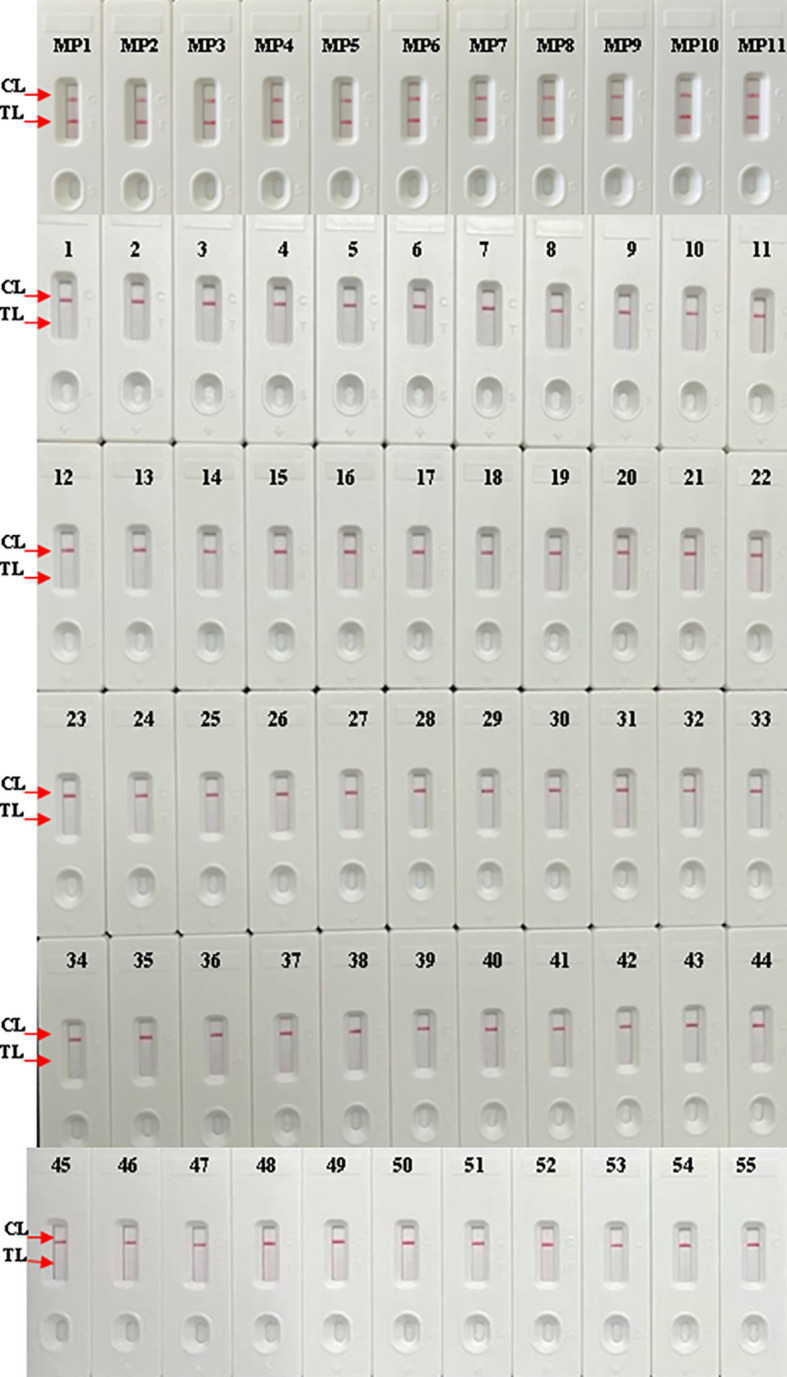
Analytical specificity of *M. pneumoniae*-LAMP-LFB assay. The LAMP-LFB assay was conducted using genomic DNA of different pathogens as templates. Biosensor MP1-MP11, *M. pneumoniae* strains; biosensors 1-44 represented the other bacterial strains except *M. pneumoniae* shown in **Table 2**. CL, control line; TL, test line.

The LAMP-LFB assay was established along with the optimization of primer sets and reaction temperature in this study. A total of eight temperatures were tested and 63°C proved to be the optimal one. Owing to the six oligonucleotide primers located at different regions of the CARDS toxin gene, the LAMP reaction in this report was revealed to be highly specific as currently formulated (Waites et al., 2017). Among the 66 tested pure cultures, only the 11 *M. pneumoniae* strains were confirmed to be positive by the LAMP-LFB assay established in this report, while the others were all negative and without any cross-reactivity (**Figure 6**), suggesting a specificity of 100% for this new LAMP-LFB assay. In terms of specificity, LAMP-LFB assay is superior to serology tests and more accurate and more reliable for the diagnosis of *M. pneumoniae* infection.

After amplification at 63°C for 40 min, the LoD of the LAMP-LFB assay targeting the CARDS toxin gene was examined as 50 fg per reaction (~12 copies) in pure cultures in this study, which is the same as that of MCDA–LFB (multiple cross displacement amplification coupled with a nanoparticle-based lateral flow biosensor) assay (Wang et al., 2019), but significantly lower than that of another LAMP-LFB assay with P1 gene as the target (Wang et al., 2019), indicating its higher sensitivity. In the application evaluation of clinical specimen, more suspected samples were detected (50%) by LAMP-LFB assay compared to those by real time-PCR (47%). According to the kit manual, the LoD of the real time-PCR assay used in this report is 400 copies, which equals about 1.6pg DNA template, while the *M. pneumoniae*- LAMP-LFB assay we conducted displayed better sensitivity with LoD of 50fg DNA template. So the LAMP-LFB assay yielded higher positivity rate than that of the real-time PCR assay in the clinical samples. Besides the lower LoD of the real-time PCR kit, the presence of some inhibitors specific to real-time PCR may also contribute to the lower positive rates of the real-time PCR detections. Although no significant statistical difference was found in the method in this report, the trait of more sensitivity of LAMP-LFB assay will be further validated if more clinical samples are involved, just as the previous report targeting the specific P1 gene of *M. pneumoniae* (47.8% versus 31.6%) (Wang et al., 2019). What’s more, the superiority of LAMP-based methods against culture, serology, conventional, and real-time PCR has been evaluated in numerous studies and countries with different detection formats (Gotoh et al., 2012; Ratliff et al., 2014; Petrone et al., 2015). Nowadays, large amounts of LAMP-based tests for the detection of *M. pneumoniae* have already sprung up and become commercially available, such as the Loopamp *Mycoplasma pneumoniae* DNA amplification kit (Eiken Chemical, Tokyo, Japan) (Matsuda et al., 2013) and the *illumigene* Mycoplasma (Meridian BioScience, Inc., Cincinnati, OH, USA) (Ratliff et al., 2014), indicating the promising future of this technology. In our manuscript, the LAMP products were judged by LFB test, which can visually, rapidly, and objectively indicate the results without any extra instrument in only 2 min. The optimized primers we used showed marked reliability, selectivity, and sensitivity. The lower cost of the LAMP-LFB assay could benefit extensive application prospects in resource-limited laboratories.

In conclusion, a new LAMP-LFB assay targeting the CARDS toxin gene for *M. pneumoniae* detection was successfully established in this study. This assay is proven to be simple, fast, sensitive, specific, and cheap, and has the potential to be widely and effectively used in the surveillance and detection of *M. pneumoniae* infection in the early stage in medical institutions and even in rural areas.

## Data Availability Statement

The original contributions presented in the study are included in the article/supplementary files. Further inquiries can be directed to the corresponding authors.

## Ethics Statement

The studies involving human participants were reviewed and approved by The studies involving human participants were reviewed and approved by the ethic committee of Capital Institute of Pediatrics (Ethical approval number: SHERLL2021010). The patients/participants [legal guardian/next of kin] provided written informed consent to participate in this study. Written informed consent to participate in this study was provided by the participants’ legal guardian/next of kin.

## Author Contributions

YW conceived study. YW and XC supervised this study. FX and CS performed the experiments. FX and JZ analyzed the data and drafted the manuscript. CS, FX, JF, XH, NJ, and ZX contributed to the reagents and materials. BZ did clinical guidance. YW conducted the software. YW and JZ revised the manuscript. All authors contributed to the article and approved the submitted version.

## Funding

This study was funded by Beijing Nova Program (Z211100002121042), National Key Research and Development Program of China [Grant Nos. 2021YFC2301101 (Yi Wang), 2021YFC2301102 (Yi Wang)] and the Research Foundation of Capital Institute of Pediatrics (Grant No. PX2021050).

## Conflict of Interest

The authors declare that the research was conducted in the absence of any commercial or financial relationships that could be construed as a potential conflict of interest.

## Publisher’s Note

All claims expressed in this article are solely those of the authors and do not necessarily represent those of their affiliated organizations, or those of the publisher, the editors and the reviewers. Any product that may be evaluated in this article, or claim that may be made by its manufacturer, is not guaranteed or endorsed by the publisher.
